# High-multiplex tissue imaging in routine pathology—are we there yet?

**DOI:** 10.1007/s00428-023-03509-6

**Published:** 2023-02-09

**Authors:** Jakob Einhaus, Alexander Rochwarger, Sven Mattern, Brice Gaudillière, Christian M. Schürch

**Affiliations:** 1 Department of Anaesthesiology, Perioperative & Pain Medicine, Stanford University School of Medicine, Stanford, CA, USA; 2 Department of Pathology and Neuropathology, University Hospital and Comprehensive Cancer Center Tübingen, Tübingen, Germany

**Keywords:** High-multiplex tissue imaging, Tumor microenvironment, Pathology, Cancer, Molecular tumor board, Review

## Abstract

High-multiplex tissue imaging (HMTI) approaches comprise several novel immunohistological methods that enable in-depth, spatial single-cell analysis. Over recent years, studies in tumor biology, infectious diseases, and autoimmune conditions have demonstrated the information gain accessible when mapping complex tissues with HMTI. Tumor biology has been a focus of innovative multiparametric approaches, as the tumor microenvironment (TME) contains great informative value for accurate diagnosis and targeted therapeutic approaches: unraveling the cellular composition and structural organization of the TME using sophisticated computational tools for spatial analysis has produced histopathologic biomarkers for outcomes in breast cancer, predictors of positive immunotherapy response in melanoma, and histological subgroups of colorectal carcinoma. Integration of HMTI technologies into existing clinical workflows such as molecular tumor boards will contribute to improve patient outcomes through personalized treatments tailored to the specific heterogeneous pathological fingerprint of cancer, autoimmunity, or infection. Here, we review the advantages and limitations of existing HMTI technologies and outline how spatial single-cell data can improve our understanding of pathological disease mechanisms and determinants of treatment success. We provide an overview of the analytic processing and interpretation and discuss how HMTI can improve future routine clinical diagnostic and therapeutic processes.

## Introduction

Antibody-based immunohistological methods have enabled great advances in the field of biosciences and in our understanding of pathological processes on a molecular and cellular level. Conventional immunohistochemistry (IHC) and immunofluorescence have been immensely influential in deciphering the phenotypic and functional architecture of tissue in health and disease. In surgical pathology, immunoassays are routinely implemented as auxiliary methods for in situ detection of single biomarkers and protein expression patterns on fresh-frozen or formalin-fixed, paraffin-embedded (FFPE) tissue sections [1]. The gained information significantly guides diagnostic processes, therapeutic decision-making, and patient risk stratification. For example, the examination of cell cycle regulators (e.g., p16, p53, bcl-2), growth stimulatory axes (e.g., estrogen and progesterone receptors, members of the epidermal growth factor receptor family such as EGFR and HER2), and immune checkpoint molecules including PD-1 and PD-L1 has added to the granularity of cancer classification systems and influences treatment choices [2, 3].

In recent years, flow- and droplet-based single-cell technologies, capturing gene transcription (single-cell RNA sequencing) and protein expression (multiparametric flow cytometry, mass cytometry), have promoted further efforts to increase our understanding of complex and heterogeneous pathological states on a single-cell level. With the rise of high-resolution, high-multiplex tissue imaging (HMTI) platforms, immunohistological investigation of spatial tissue functionality and organization is combined with single-cell resolution [4]. As a major improvement over existing molecular histopathology methods that are mainly performed single-parametrically, these platforms extend the simultaneously captured information to a multitude of cellular subsets, including innate and adaptive immune cells, stromal cells, and tumor cells by allowing multiparametric assays and enable in-depth characterization of crucial cell–cell interactions within their respective spatial context. Different approaches have overcome the limitations of spectral overlap in conventional fluorescence techniques and increased the possible target number to more than 50 targets [5]. The produced datasets pose a challenge for the interpretation and analysis, but addressed with proper computational tools and machine learning strategies, the potential to improve patient care strategies and clinical workflows is unprecedented.

Here, we provide an overview over existing HMTI techniques and how their application can improve our understanding of disease mechanisms and the refinement of treatment strategies. We then discuss challenges of how to process, analyze, and interpret findings with these imaging approaches. Finally, we outline how HMTI can be implemented into clinical routine pathology and decision-making to improve and optimize diagnostic and therapeutic processes.

## Antibody-based high-multiplex tissue imaging methods

**Fig. 1:**
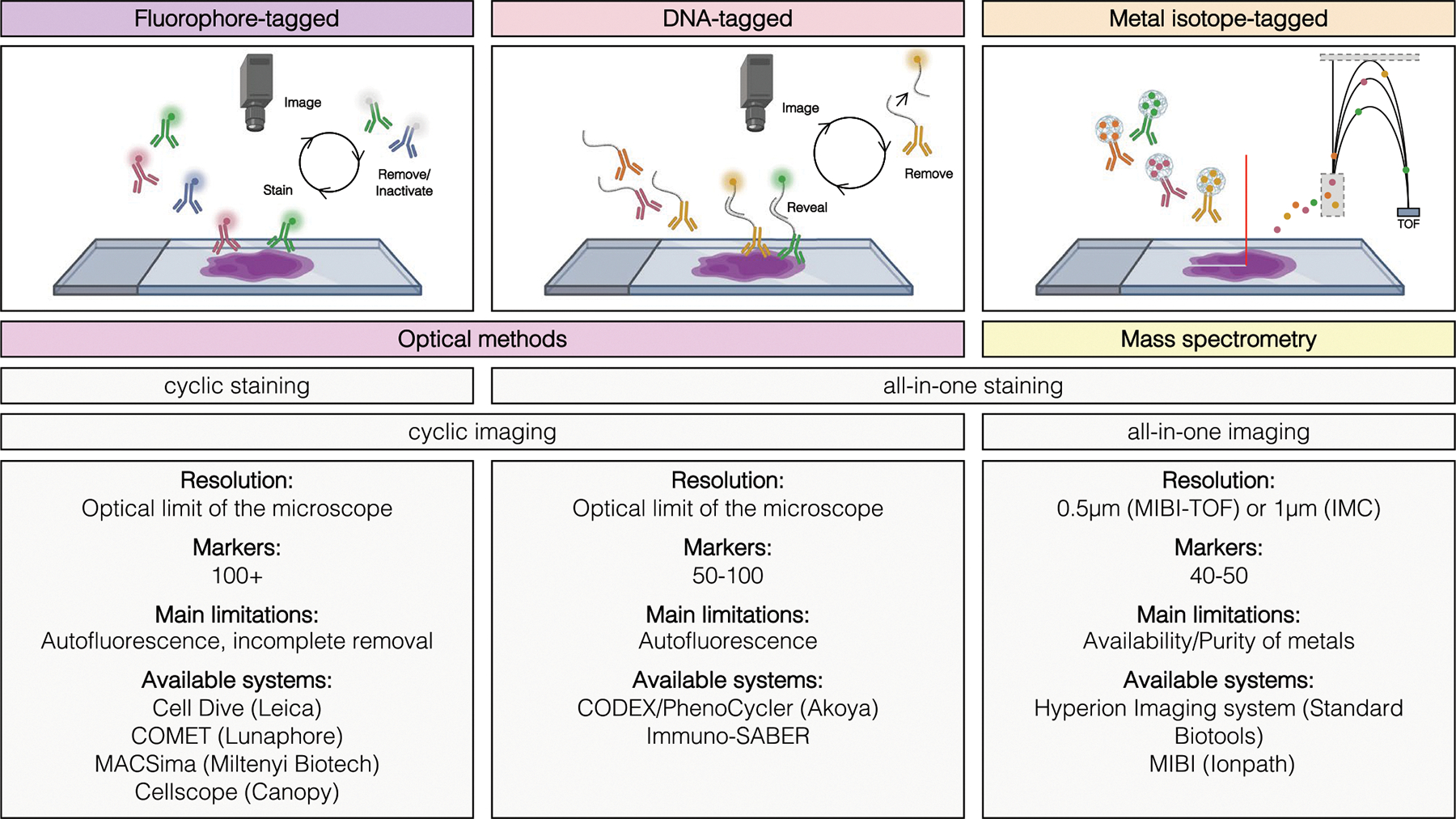
Overview over existing antibody-based high-multiplex tissue imaging techniques. Existing antibody-based HMTI approaches use fluorophore-, DNA-, or metal-tagged antibodies for target identification. Various systems with different characteristics and limitations are commercially available

While conventional antibody-based imaging techniques such as IHC and immunofluorescence are limited to two to three or four to seven markers, respectively, due to the spectral overlap limitation, multiplex imaging approaches overcome this obstacle in a variety of ways [6, 7]. Antibody-based HMTI approaches differ regarding immunolabeling methods and the corresponding tag detection. The most established methods use DNA oligonucleotide–tagged, fluorophore-tagged, or metal-tagged antibodies. Methods using fluorescence often implement cyclic staining and detection to avoid spectral overlap, while metal-tagged technologies using mass spectrometry have lower interference and can acquire samples all-in-one (Fig. 1).

## Fluorophore-tagged antibody-based HMTI methods

Multiplex fluorescence imaging of more than 90 markers within a single tissue sample was described as early as 2006 [8]. The underlying concept of the technologies that evolved from this technique is mostly referred to as multiplexed immunofluorescence (MxIF) or tissue-based cyclic immunofluorescence (t-CyCIF). These fluorescence-based HMTI approaches extend conventional fluorescence microscopy by cyclical staining protocols [9], allowing multi-marker panels through successive bleaching or removal of the fluorescent dye–tagged antibodies after each staining and imaging cycle. The cyclic incubation with bleaching reagents and antibodies overcomes the limitation of spectral overlap of fluorochromes but prolongs the imaging process proportionally to the number of markers included. Compared to other HMTI modalities, fluorescence-based technologies are the most established due to the broad availability of knowhow, instruments, and reagents [10]. Commercially available, automated platforms for multiplex immunofluorescence with up to eight markers, such as Vectra^®^ Polaris^™^, have proven great value and reliability as high-throughput technologies in predictive studies of patient outcomes [11, 12]. Development of similar, widely available, and automated HMTI platforms with compatible panels could offer consistent standardization and reliable intersample comparability for routine clinical application. Limitations of the fluorophore-tagged antibodies include autofluorescence, incomplete bleaching, and tissue destruction [5].

## DNA-tagged antibody-based HMTI methods

Technologies that use DNA-tagged antibodies, such as in CO-Detection by indEXing (CODEX) or Immunostaining with Signal Amplification By Exchange Reaction (ImmunoSABER) [13, 14], have more recently evolved as alternative HMTI platforms. The core principle of these technologies is the combination of immunostaining with DNA oligonucleotide–linked antibodies and sequential readout using complementary DNA oligonucleotides bound to fluorophores. After immunostaining of the tissue samples with currently up to 60 DNA-tagged antibodies, fluorophore-tagged complementary DNA sequences are cyclically hybridized and washed out of the stained tissue, thereby rendering a subset of the DNA-linked antibodies visible during a multicyclic run. After hybridization within each cycle, imaging with a fluorescence microscope is performed. Nuclear staining with DAPI or Hoechst is performed in each cycle for cellular alignment.

While the first CODEX system used primer extension by DNA polymerase and fluorophore-tagged dNTP analogs for barcode detection [13], current CODEX systems use fluorophore-tagged complementary DNA sequences [15, 16]. In the case of ImmunoSABER, antibodies are linked to a short DNA sequence called bridge strand. The respective complementary DNA strand is independently extended to a controlled length with a short repetitive sequence using primer exchange reactions, producing so-called concatemers [17]. After hybridization of the concatemer with the antibody-linked bridge strand, the repetitive sequences of the concatemer serve as complementary binding sites for fluorophore-labeled imager strands. The utilization of repetitive sequences with definable length in the ImmunoSABER system allows for a range of signal amplification. Within the CODEX system, signal amplification has been described by means of peroxidase-conjugated secondary antibodies that activate tyramide-conjugated DNA barcodes [18]. Depending on available filters and the fluorescence microscope used, multiple fluorophores for different DNA barcodes can be imaged simultaneously to reduce acquisition times. The panel size for multiplexed imaging with DNA-tagged antibodies is in practice limited by the amount of available non-cross-reactive DNA barcodes, underscoring the need to expand the range of suitable tags through identification and validation of a broad range of theoretical DNA sequences [19].

## Metal-tagged antibody-based HMTI methods

As an alternative to fluorescence-based HMTI approaches, mass spectrometry (MS)–based HMTI technologies detect the isotope mass of metal-tagged antibodies. The MS-based cytometry by time-of-flight (CyTOF) platform offers the advantage to simultaneously detect a high number of isotope tags with minimal spectral interference and has been established for single-cell suspension cytometry over the last decade [20]. Imaging mass cytometry (IMC) [21] extends the use of the CyTOF system by attaching a tissue imaging instrument to the sample inlet line. Using a high-energy laser beam to ablate the tissue, the tissue is vaporized pixel by pixel and conducted into the CyTOF system as an ion cloud for MS analysis. The focus of the laser determines the imaging resolution to currently 1 μm, and acquisition times lie around 60 min per mm^2^. On the other hand, multiplexed ion beam imaging (MIBI) [22] is a standalone MS-based imaging system that uses a primary ion beam to release a secondary ion cloud from the stained tissue for MS analysis. The ion beam can be focused to a minimal spot size of 250 nm, and slides can be rescanned repetitively; however, this comes at the cost of longer acquisition times. In both MS-based technologies, primary antibodies are conjugated to polymers loaded with heavy metal isotopes that do not naturally occur in organic tissues, mainly isotopically purified lanthanide metals. This allows for the simultaneous detection of 40 + biomarkers in FFPE tissues with low background signal and high sensitivity, no autofluorescence of tissue, and minimal spectral overlap. However, this prerequisite currently limits the panel size to around 50 markers, for which reagents are commercially available. The investigated tissue can be stained with a master mix of primary metal-conjugated antibodies without the need of cyclic staining or acquisition. Additionally, MS-based technologies allow the combination of multimodal target detection, as demonstrated for the use of protein and RNA-detecting antibodies [23].

## Synergistic potential with spatial transcriptomics and spatial metabolomics

HMTI methods extend far beyond antibody-based tissue imaging. Other single-cell or high-throughput technologies, such as transcriptomics or metabolomics, have also been adopted to include spatial resolution. Early approaches in spatial transcriptomics by means of fluorescence in situ hybridization (FISH) allowed the identification of single-RNA molecules but were limited in the amount of targets [24]. The introduction of novel technologies, such as multiplex error-robust FISH (MERFISH) [25] or sequential FISH (seqFISH +) [26], originated the technical development towards high-resolution transcriptomics by allowing measurements of hundreds to thousands of different RNA molecules on single-cell level without the need for tissue dissociation. In these technologies, RNA identification is accomplished by decoding the binary or temporal barcodes generated for each RNA molecule during the sequential hybridization and imaging cycles. First, target RNA molecules are hybridized with encoding probes that are flanked by readout sequences. Fluorophore-tagged readout probes that bind to the readout sequences are then introduced to the labeled tissue in successive cycles [4]. Another commercially available RNA imaging technology is RNAscope^®^, which relies on signal amplification with branched DNA constructs. Target RNA molecules are bound by pairs of Z-probes that are successively bound by target-specific preamplifier molecules. These preamplifiers offer multiple binding sites to amplifiers that are in turn bound by multiple fluorescent label probes, resulting in selective signal amplification with low background signal [27]. RNAscope^®^ technology can be applied to FFPE tissue and offers up to 12-plex imaging.

In general, the respective HMTI methods are not necessarily confined to the detection of a single type of target molecule. Recent concepts and protocols have describe simultaneous or successive imaging of both proteins and RNA in tissue, e.g., by including RNAscope^®^-derived branched DNA constructs with metal-tagged label probes in IMC panels [23], or combining CODEX and RNAscope^®^ [28]. The SM-omics platform achieves further integration of synergistic readouts, as it combines antigen detection with DNA-barcoded antibodies with spatial transcriptomics in one analytic system [29]. Additionally, novel technologies such as CosMx^™^ [30] are directly applicable for both RNA and protein imaging.

Another emerging spatial omic technology is spatial metabolomics. Technological improvements have recently increased the resolution of metabolomic imaging approaches to allow the identification of peptides, lipids, and drugs on a single-cell level [31]. Mostly relying on MS-based metabolite identification, e.g., matrix-assisted laser desorption/ionization, spatial metabolomic technologies aim to differentiate cell populations within tissue samples with spatially distinct, corresponding metabolomic profiles.

While these technologies are not routinely used in surgical pathology, metabolomic imaging has been shown to differentiate tumor from normal tissue based on distinct metabolic profiles [32], to aid patient stratification by identifying tumor subpopulations [33], and to predict treatment response to neoadjuvant therapy [34]. As an alternative to MS-based metabolomics, Raman spectroscopy offers metabolomic spatial profiling by measuring inelastic scattering of photons. The sample is illuminated with monochromatic light, and after interaction of the light photons with the tissue, the detected Raman scattering, whose characteristics depend on the spatial occurrence of distinct chemical bonds, allows to draw conclusions about the metabolomic profile of the tissue [35]. While Raman spectroscopy is clinically most often applied in a surgical context [36, 37], the recent development of Raman-active nanoparticles has improved the usefulness of Raman microscopy as a method of multiplex metabolomic imaging in both fixed and live cells [38, 39].

In summary, spatial profiling of transcriptome and metabolome complement existing antibody-based HMTI approaches, and the integration of spatial multiomic information into clinical decision-making will aid many fields to move towards personalized medicine [6].

## Machine learning approaches as novel predictive and analytic tools for HMTI data

**Fig. 2:**
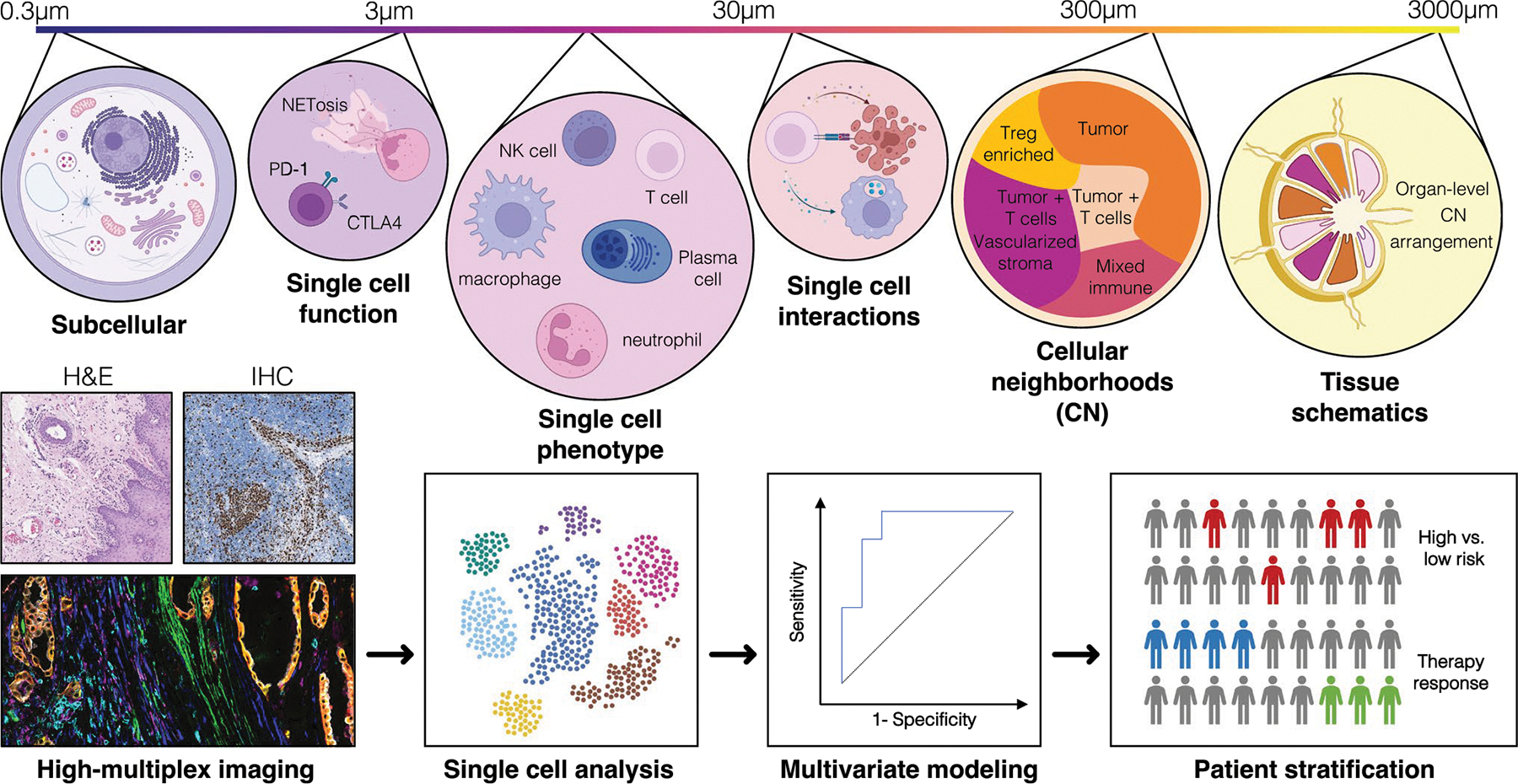
Levels of information and analysis workflow for high-multiplex tissue imaging approaches. HMTI data contain information that can be analyzed on multiple levels. They allow gathering of subcellular information close to the optical limit of microscopy (~ 0.3 μm per pixel), functional aspects of single cells, single-cell phenotypes and cell–cell interactions, as well as superordinate levels of the organization, such as cellular neighborhoods [76] or tissue schematics [84]. In contrast to hematoxylin and eosin (H&E) staining or single parametric IHC, HMTI allows generating single-cell datasets for multivariate modeling, thereby enabling accurate risk stratification (high vs. low) or prediction of therapy response. The CODEX image (bottom left) was reproduced from [76] under a Creative Commons CC-BY 4.0 license

HMTI techniques pose unique challenges for data analysis and interpretation. For biologically meaningful interpretation, the produced raw data, i.e., pixel intensities for each acquired marker, need to be transformed into single-cell data through cell segmentation. Various segmentation tools have emerged to define cell boundaries and assign pixels to the respective cell, both supervised, human-trained machine learning algorithms (e.g., RetinaMask, StarDist, Ilastik) [40–42] as well as unsupervised, automated deep learning models (e.g., FeatureNet, CellPose, CellSeg, Mesmer) [43–46]. The resulting single-cell dataset includes marker intensity values, meta-variables such as object area or eccentricity, as well as spatial X/Y coordinates (centroids) for each cell. By identifying cell populations through one of various cell phenotyping approaches [20, 47, 48], spatial abundance, activity, and interaction patterns become interpretable for univariate and regression analysis of outcome, or they can serve as features in multivariate modeling approaches with classical machine learning algorithms (Fig. 2). While conventional machine learning requires a preprocessed dataset of derived features, deep learning approaches unite the steps of feature extraction and classification, using neural networks to produce predictive models. Deep learning can prove advantageous particularly in large datasets, independent of dimensionality of the dataset: using H&E-stained images as training data, Kather et al. demonstrated how neural networks can offer highest accuracy in the prediction of tumor characteristics [49] or outcomes [50] from ubiquitously available digital histology slides.

## Clinically relevant pathology insights uncovered by high-multiplex tissue imaging

The described HMTI platforms offer unprecedented insight into the spatial single-cell structure of pathologies. Tumor pathology has been a major focus in initial applications of HMTI, as interindividual and intraindividual tumor heterogeneity complicate diagnostic and therapeutic optimization and HMTI provides an avenue to globally capture these complex tissues. Specifically, thorough characterization of the complex cellular interactions within the tumor microenvironment (TME) [51] represents a field of highest interest. In the immune TME, the identification of novel biomarkers for immunotherapy response and the detection of new molecules or mechanisms that can be targeted with future innovative treatments could drastically improve patient outcomes. Other complex pathologies with a distinct histological footprint, such as autoimmune or infectious diseases, are also starting to reveal novel biology from HMTI studies [52]. However, widespread adoption of these technologies is still in its infancy and biological discoveries are yet to be clinically implemented. In the following paragraphs, we will present existing examples of clinically relevant information uncovered with HMTI approaches for a variety of relevant conditions, thereby demonstrating how spatial single-cell analysis can help define molecular biomarkers for clinical applications.

### Breast cancer

Important classifiers in the pathological evaluation of breast cancer, one of the most frequent of all human tumor types, are immunohistological parameters: HER2 status and the expression of estrogen and progesterone receptors are routinely used to determine breast cancer subtype and to guide optimal treatment plans accordingly [53–55]. In the case of HER2 positivity, trastuzumab, a monoclonal antibody, should be considered as effective neoadjuvant treatment option before surgery and hormone receptor status influences the adjuvant therapy regimen with endocrine therapy [56]. While currently available treatment regimens continue to change, particularly with the introduction of the antibody–drug conjugate trastuzumab-deruxtecan for HER2-low expressing tumors [57], immunodetection methods serve as established tumor subclassification tools to analyze tumor cell phenotype and functional state. But not only the tumor cell properties define the success of therapies and the prognosis of breast cancer. The TME, i.e., the mutual influence of cancer cells and their surrounding cells, such as innate and adaptive immune cells, fibroblasts, or other stromal cells, plays a critical role in the development and progression of solid tumors [51, 58]. In this context, HMTI approaches offer the advantage of enabling the simultaneous detection of multiple cell subsets, even in combination with functional markers, by allowing for comprehensive antibody panels. In triple-negative breast cancer, Keren et al. could distinguish three different archetypes of tumor-immune interaction, namely cold, compartmentalized, and mixed tumors [59]. This difference in spatial architecture was accompanied by contrasting expression patterns of immunoregulatory proteins on the single-cell level. When correlated to outcome, patients with compartmentalized tumors had a significantly reduced survival compared to patients with mixed tumor architecture. Similarly, single-cell analysis of the breast cancer TME has been used to identify tumor and microenvironment communities based on cellular composition, and the presence of communities differentiate tumor pathology subgroups with distinct clinical outcomes [60]. As an example, this large patient cohort with 300 breast cancer samples revealed that high levels of hypoxic, p53^+^EGFR^+^, or proliferative markers on tumor cells are linked to poor survival. Lastly, HMTI can also inform about the state of anticancer immune mechanisms: the presence of checkpoint molecules and indicators of T cell exhaustion can be determined across large patient cohorts, and the derived patient classification might aid matching of precision medicine approaches to individual tumor subclasses [61].

### Malignant melanoma

The treatment of malignant melanoma has changed dramatically over the last decade after the introduction of the first-ever FDA-approved checkpoint inhibitor ipilimumab in 2011. The following rise of immunotherapies has significantly improved the prognosis of malignant melanoma, even in advanced and metastatic cases [62]; however, treatment success remains variable between individual patients and accurate prediction of the response to immunotherapy is lacking [63]. Analysis of PD-1/PD-L1 and infiltration of CD8^+^ T cells represent potential biomarkers but need to be contextualized for reliable interpretability [64]. Here, HMTI can help to capture complex patterns of T cell distribution, functionality, and exhaustion. A recent study focused on the association between immune infiltrates and immunotherapy response [65]. Both a higher abundance of proliferating, antigen-experienced cytotoxic T cells in the immune TME of malignant melanoma and closer spatial contact between melanoma and antigen-experienced T cells were good indicators of favorable immunotherapy response. Additionally, HMTI can elucidate complex mechanisms to explain clinical outcomes. Combining RNA and protein detection through IMC, Hoch et al. analyzed specialized chemokine milieus in metastatic melanoma [66]. Interestingly, the analysis showed that chemokine expression patterns in T cells were indicative of T cell exhaustion, while CXCL13-producing T cells were crucially involved in the formation of tertiary lymphoid structures. Ultimately, the data pointed to a chemokine-regulated, underlying mechanism for both T cell function and tertiary lymphoid structure formation, which correlates with immunotherapy responses in multiple tumor types [67–69].

### Colorectal cancer

Colorectal cancer represents the third leading cause of cancer death and the fourth most diagnosed cancer worldwide [70]. Recent studies have emphasized the prognostic value of assessing immune cell infiltrates, specifically regulatory T cells and CD8^+^ T cells, within colorectal cancer samples [71–73]. Overall, higher T cell presence in colorectal cancer samples was associated with better outcomes, while immune-cold tumors had a worse prognosis. Hereby, automated scores based on immune cell classification with multi-marker panels also provide high accuracy for patient stratification and treatment response assessment [74]. HMTI can now combine immune feature analysis with known histological parameters to improve our understanding of critical aspects of tumor immune interplay that functionally determines outcome and survival. In colorectal cancer, Crohn’s-like reaction describes a type of immune TME with the presence of tertiary lymphoid structures without evidence of prior Crohn’s disease and is associated with improved survival [75]. We used CODEX HMTI to dissect the tissue architecture of tumors displaying a Crohn’s-like reaction immune TME in comparison to samples with diffuse inflammatory infiltration without tertiary lymphoid structures in the TME. By introducing the concept of cellular neighborhoods and defining functional regions within the immune TME based on cell type stoichiometry, this approach demonstrated how cell type abundance can inform about patient prognosis when analyzed within the spatial context of its surroundings [76]: the frequency of PD-1^+^CD4^+^ T cells in granulocyte-enriched cellular neighborhoods was associated with improved survival in patients with diffuse inflammatory infiltration-type immune TME, and could be used to stratify patients into more specific subgroups. These findings confirm the potential of HMTI, in combination with sophisticated analysis tools, to increase the access to valuable information for individualized diagnosis, prognosis, and treatment decisions.

### Autoimmunity/infectious disease

This principle also applies to autoimmune and infectious diseases. In ulcerative colitis, assessing cellular interactions within their spatial context revealed evidence of sex-dependent differences in therapeutic response to TNF inhibitors [52]. With no existing clinical biomarkers for TNF inhibitor response so far, this study demonstrated that HMTI, paired with neural network modeling, has potential as a precision medicine tool that could complement other predictive diagnostic modules such as radiologic imaging or laboratory tests.

In other applications, HMTI offers unprecedented insight into pathogenetic mechanisms and new therapeutic perspectives. Damond et al. derived a timeline of disease progression for type 1 diabetes mellitus from imaging of pancreatic islets with IMC and a pseudotime data analysis approach [77]. This data analysis method represents a framework for further research exploring the pathogenesis and progression of chronic disease not necessarily limited to type 1 diabetes mellitus. Lastly, HMTI can uncover unexpected biological features, as described by McCaffrey et al. in a study using MIBI to analyze granulomas in patients with tuberculosis [78]. In tuberculosis granulomas, immunoregulatory cell subset distribution and marker expression patterns resembled immune evasion mechanisms observed in the immune TME and could contribute to chronic bacterial persistence. Additionally, these findings were also reflected in transcriptomic expression dynamics in matching peripheral blood samples, pointing to a systemic immune signature of immunosuppression in tuberculosis. This example illustrates the relevance of integrative studies that combine HMTI data with systemic omic modalities in order to capture local and systemic aspects of complex diseases globally.

## Future integration of high-multiplex tissue imaging approaches into the routine clinical workflow—bridging the gap between discovery platform and clinical routine

**Fig. 3:**
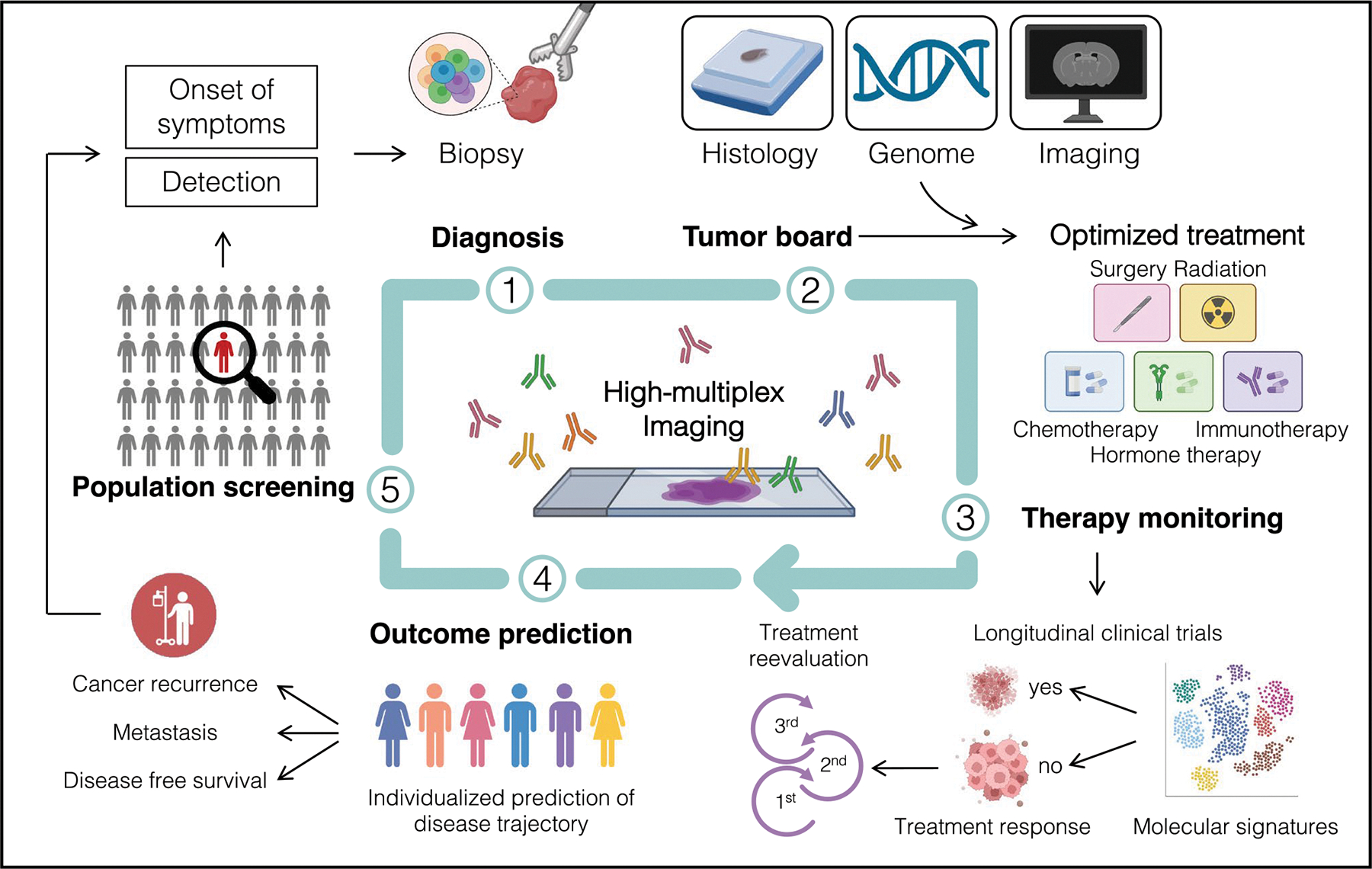
Integration of high-multiplex tissue imaging into an idealized future clinical workflow and decision-making. HMTI can offer high-yield information at multiple steps along existing clinical workflows. For instance, in tumor patients, HMTI provides an accurate diagnostic tool, informs clinical decision-making by molecular tumor boards, aids monitoring therapy response as well as outcome prediction, and strengthens existing screening approaches

HMTI technologies are becoming increasingly available in laboratories, companies, and pathology departments. Due to their novelty and complex challenges in data analysis, their integration into clinical workflows for patient care, unlike well-established single-parameter immunohistological assays, is still in its infancy and not yet implemented. However, HMTI technologies with single-cell resolution hold the promise of enabling highly accurate patient stratification and personalized medicine, by making complex tissue architectures and cellular neighborhoods accessible as predictive biomarkers for clinical translation. In a first step, exploratory studies along existing patient management paths are necessary to characterize single-cell architecture patterns indicative of diagnoses, treatment responses, and outcomes (Fig. 3). The adoption of tissue microarrays as an established tissue resource in HMTI studies has shown that small tissue areas contain an unmatched plethora of information that suffices for accurate predictive models. Hence, HMTI technologies represent ideal analysis tools for small clinical biopsies that could be gathered with minimal invasiveness, to maximize information content from the available tissue. Of note, large pathology institutes usually have 300 or more standardized IHC antibodies available, which could theoretically be combined into three to five HTMI panels to streamline immunohistochemistry. Gradually, prospective implementation of HMTI into clinical workflows could provide a powerful diagnostic tool that adequately addresses tissue heterogeneity found in many pathologies. Central applications of these assays could be the optimization and personalization of treatment strategies, treatment response monitoring, and accurate predictions of the outcome. Irmisch et al. provide an example of the integration of multi-omic investigations into the clinical decision-making, specifically by a multidisciplinary tumor board [79].

**Fig. 4:**
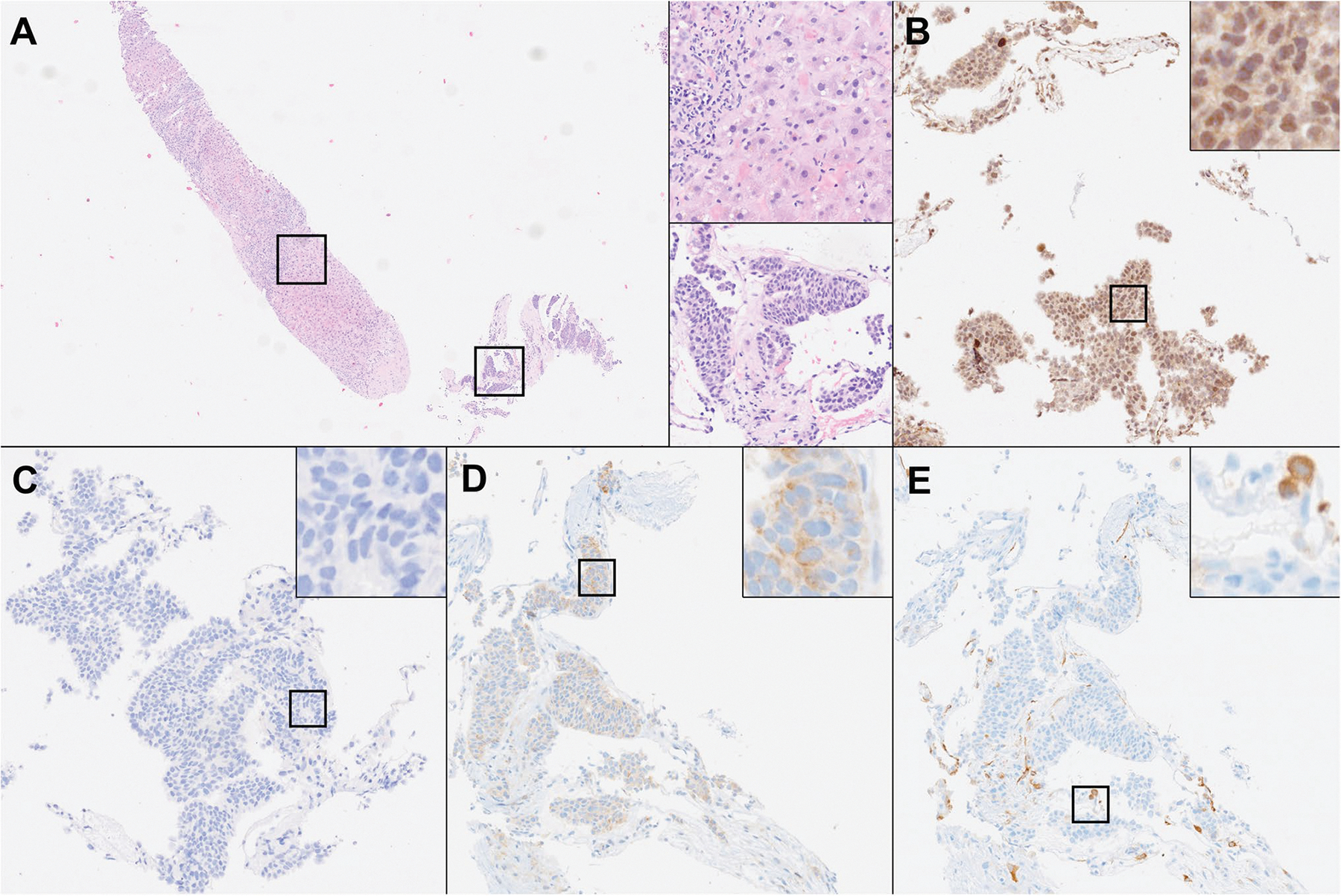
Core needle biopsy from a hepatic metastasis of a neuroendocrine tumor of unknown primary site. **A** After routine diagnostics and molecular analysis of this exemplary patient case, little tissue remains on the paraffin block (H&E stain). Upper Inset (200 ×): non-neoplastic liver tissue, making up the largest portion of the core needle biopsy. Lower inset (200 ×): tumor with small to medium-sized tumor cells with monotonous nuclei and nest-like growth pattern. **B**–**E** Single parametric IHC stains for phosphorylated (p)AKT(Thr308) (**B**), pAKT(Ser473) (**C**), pmTOR (**D**), and pS6 (**E**) are performed to quantify signaling activity in the tumor and to support therapeutic decision-making in cases with molecular evidence of increased PI3K/ AKT/mTOR-pathway activation (200 × , insets showing the staining pattern of each antibody at 800 ×)

At present, our molecular tumor board at the University Hospital Tübingen, Germany, uses extensive genetic tumor analysis [80], in most cases supported by IHC, as the basis of clinical decision-making [81, 82]. Most patients discussed at the molecular tumor board have already received multiple lines of therapy and are left with limited therapeutic options. Therefore, sampling of the tumor needs to be reiterated as previous exposure to (multiple) different substances might have changed and shaped the tumor’s genetic and functional profile, and the TME. Screening for a plethora of targetable genetic alterations, including mutations, deletions, inversions, or translocation informs about the suitability of targeted treatment approaches, such as tyrosine kinase inhibitors or monoclonal antibodies. However, accurate prediction of functional consequences of genetic alterations remains challenging and genetic evaluation should be combined with pathway analyses of actual upstream and downstream signaling activity, e.g., through IHC for protein phosphorylation events (Fig. 4) [81]. In practice, the tissue remaining for IHC testing is often extremely sparse, due to several factors. First, tumors are mostly sampled with minimally invasive, fine-needle biopsies to help reduce the interventional risk and shorten hospital stays, thereby limiting the amount of available tissue in the first place. Second, necrosis, fibrosis, and non-neoplastic tissue can make up large portions of a biopsy. Lastly, routine diagnostic steps are required to confirm the diagnosis and to rule out the possibility of a secondary neoplasm.

Here, HMTI technologies offer a solution to these practical challenges that limit the information available for clinical decision-making by the interdisciplinary tumor board team, including clinicians, pathologists, geneticists, and molecular biologists. By generating a large amount of information from a small tissue sample, HMTI can provide a comprehensive overview over the functional states of tumor cells and abate the need for additional biopsies. Additionally, multiplex antibody panels are ideally suited when encountering tumor with great heterogeneity, in which signaling activity might be variable based on location and comparisons between regions, e.g., the invasive border and the tumor core, might be informative [83]. In large studies as well as clinical use, on-slide control samples are crucial to ensure intersample comparability and control for staining and sample quality across patients. Finally, HMTI can add a detailed evaluation of patient-specific characteristics of the TME, thereby increasing the efficacy of the cancer (immuno)therapy selected for each individual patient, improving outcomes, and reducing unnecessary side effects, overtreatment, and health care costs.

### Outlook

In summary, recently developed HMTI technologies provide well-suited tools for in-depth spatial analysis of single-cell tissue architecture. By enabling large antibody panels, HMTI allows for simultaneous assessment of phenotype (e.g., cell types) and function (e.g., checkpoint molecule expression in immune cells, phosphorylation states of different tumor-promoting pathways in cancer cells) in the context of spatial localization and cell–cell interactions, thereby combining information beyond the content single-parametric immunohistochemistry. In this review, we provided an overview over existing approaches, highlighted key scientific findings from investigations using HMTI, and pointed out challenges currently present in clinical routine pathology that could be overcome with these novel technologies. Future efforts should optimize the integration of these technologies into clinical workflows to take advantage of the plethora of information that can be extracted from any clinical tissue sample. We aim to encourage investigators to seek for direct applications of HMTI in routine workflows to enable a transition towards personalized medicine.

## Data Availability

Data sharing not applicable to this article as no datasets were generated or analysed during the current study.
